# Supplementary material for: Nucleoporin Nup155 is part of the p53 network in liver cancer
Source: Nat Commun. 2019 May 14;10:2147. doi: 10.1038/s41467-019-10133-z (PMC6517424; doi:10.1038/s41467-019-10133-z)
Supplement: Supplementary file 1 — Supplementary Information [file 41467_2019_10133_MOESM1_ESM.pdf]

Supplementary Figure 1

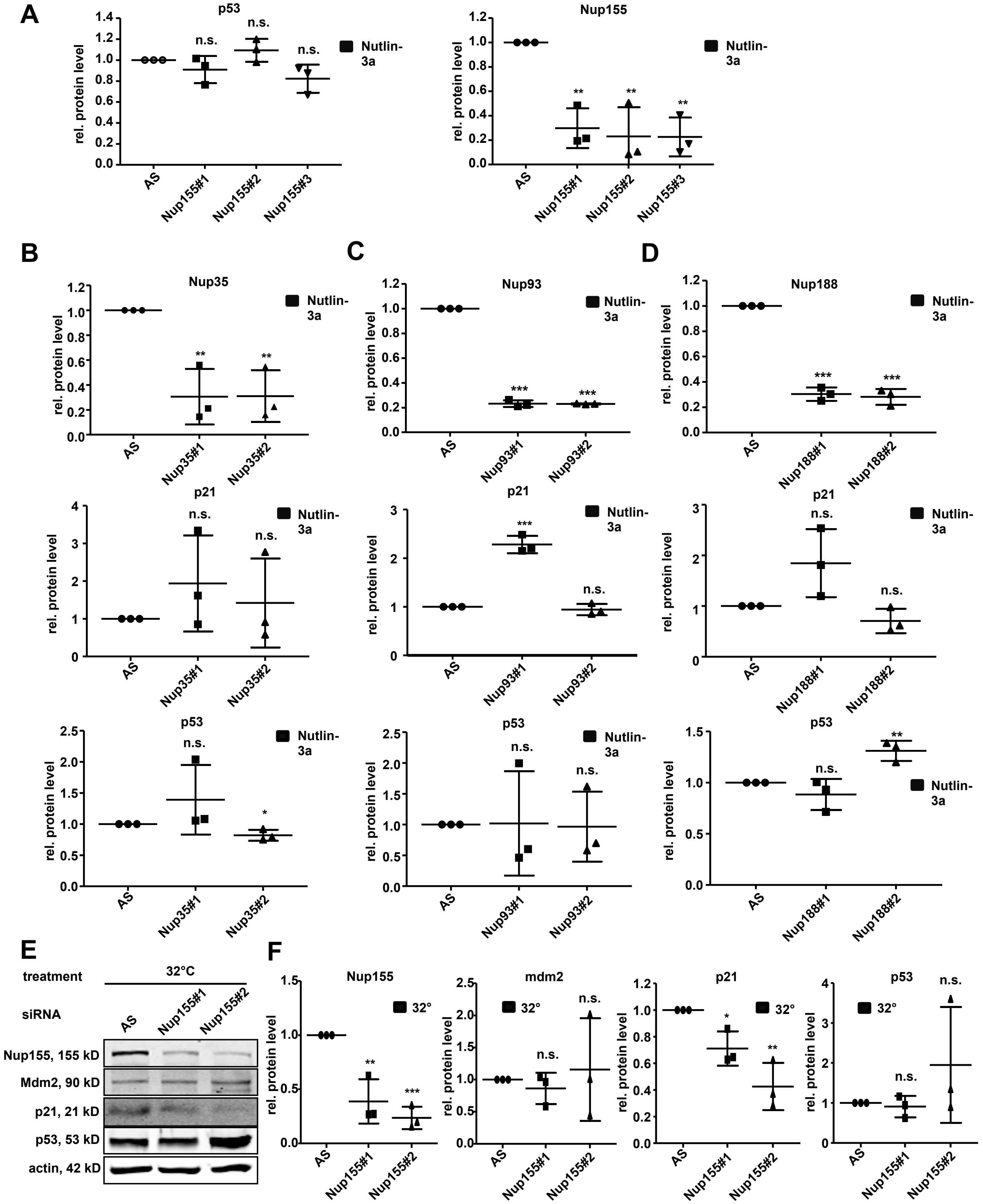

Supplementary Figure 1 A) Densitometric analyses for p53 (left panel) and Nup155 (right panel) immunoblots derived from three independent experiments (corresponding to Figure 1B) normalised to the control siRNA (AS) condition. **B)** Densitometric analyses for Nup35 (upper panel), p21 (middle panel) and p53 (lower panel) immunoblots derived from three independent experiments (corresponding to Figure 1C, left panel) normalised to the control siRNA (AS) condition. **C**) Densitometric analyses for Nup93 (upper panel), p21 (middle panel) and p53 (lower panel) immunoblots derived from three independent experiments (corresponding to Figure 1C, middle panel) normalised to the control siRNA (AS) condition. **D)** Densitometric analyses for Nup188 (upper panel), p21 (middle panel) and p53 (lower panel) immunoblots derived from three independent experiments (corresponding to Figure 1C, right panel) normalised to the control siRNA (AS) condition. **E)** Hep3B-4Bv cells harboring a temperature-sensitive mutant version of p53 that acquires wild-type function at 32°C incubation temperature were incubated at 32°C for 96h after Nup155 siRNA treatment and used for immunoblotting with the indicated antibodies. **F)** Densitometric analyses for Nup155, mdm2, p53 and p21 immunoblots derived from three independent experiments normalised to the control siRNA (AS) condition.

*$p < 0.05$, **$p < 0.01$, ***$p < 0.001$ (Student's-t-test); Data are presented as mean ± stdv. Source data are provided as a Source Data file.

Supplementary Figure 2

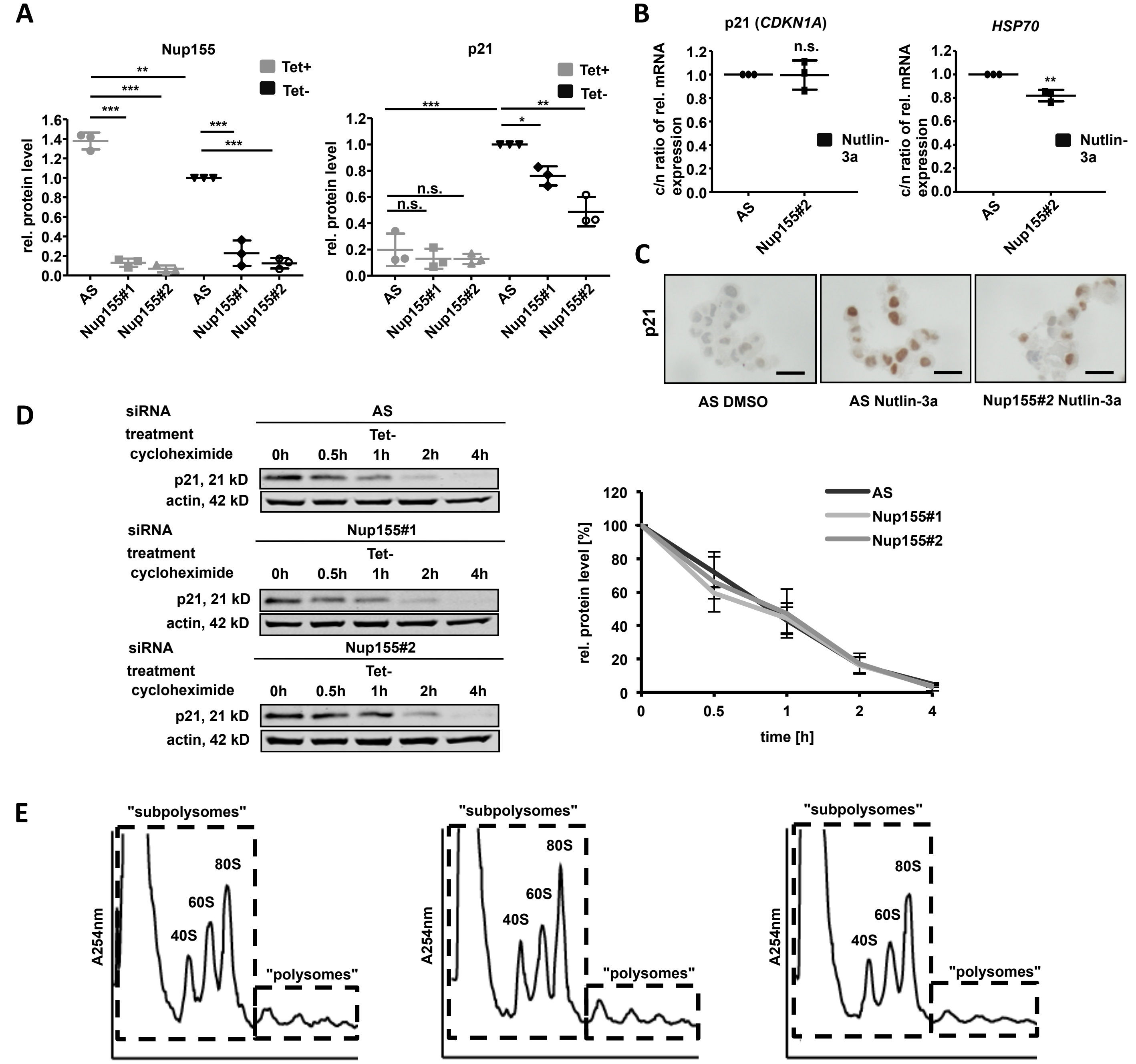

Supplementary Figure 2A) Densitometric analyses of Nup155 and p21 immunoblots derived from three independent experiments (corresponding to Figure 2A) normalised to the Tet-off control siRNA (AS) condition. **B)** HepG2 cells were treated either with control siRNA (AS) or a Nup155 siRNA#2 for 72h and Nutlin-3a was added for 24h before harvesting. p21 (*CDKN1A*) mRNA (left panel) and *HSP70* mRNA (right panel) were isolated from the nuclear and cytoplasmic fractions and measured by qRT-PCR and the cytoplasmic to nuclear ratio was calculated. Data are derived from three independent experiments and normalised to the control siRNA (AS) condition. **C)** p21 immunocytostaining of HepG2 cells treated either with control siRNA (AS) or Nup155 siRNA#2. Left panel: DMSO, middle and right panel: Nutlin-3a treatment for 24h. Scale bar = 20 µm. **D)** H24-p21 cells containing a tetracycline (“Tet-off”)-regulatable p21 expression construct were treated either with control siRNA (AS) or two Nup155 siRNAs (Nup155#1 and Nup155#2) for 72h. p21 was induced by Tet removal 96h before blocking translational elongation with cycloheximide. Cells were harvested at indicated time points and analysed by immunoblotting with the indicated antibodies (left panels). Densitometric quantification analysis of the above mentioned p21 half-life experiments (right panel) normalised to the control siRNA (AS) condition. Data are derived from three independent experiments. **E)** Polyribosome gradients corresponding to Figure 2D.
*p < 0.05, **p < 0.01, ***p < 0.001 (Student's-t-test); Data are presented as mean ± stdv. Source data are provided as a Source Data file.

Supplementary Figure 3

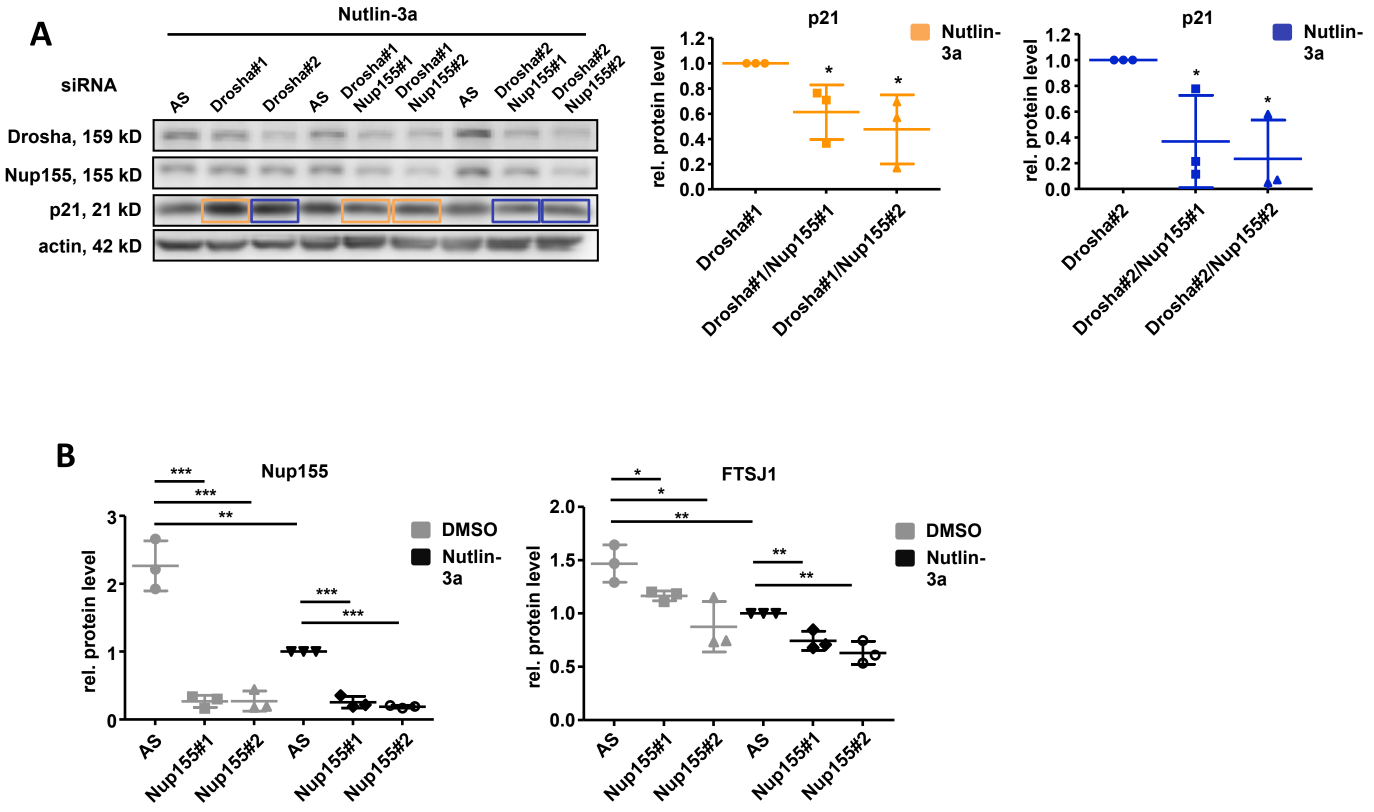

Supplementary Figure 3 A) HepG2 cells were treated either with control siRNA (AS) or two different Drosha siRNAs (Drosha#1 and Drosha#2) or in combination with two different Nup155 siRNAs (Nup155#1 and Nup155#2). Cells were harvested upon 24h of Nutlin-3a treatment and extracts were analysed by immunoblotting with the indicated antibodies (left panel). Densitometric analyses of p21 immunoblots derived from three independent experiments normalised to the Drosha#1 condition (middle panel) or normalised to the Drosha#2 condition (right panel). **B)** Densitometric analyses of Nup155 and FTSJ1 immunoblots derived from three independent experiments (corresponding to Figure 3F) normalised to the Nutlin-3a control siRNA (AS) condition.
*p < 0.05, **p < 0.01, ***p < 0.001 (Student's-t-test); Data are presented as mean ± stdv. Source data are provided as a Source Data file.

Supplementary Figure 4

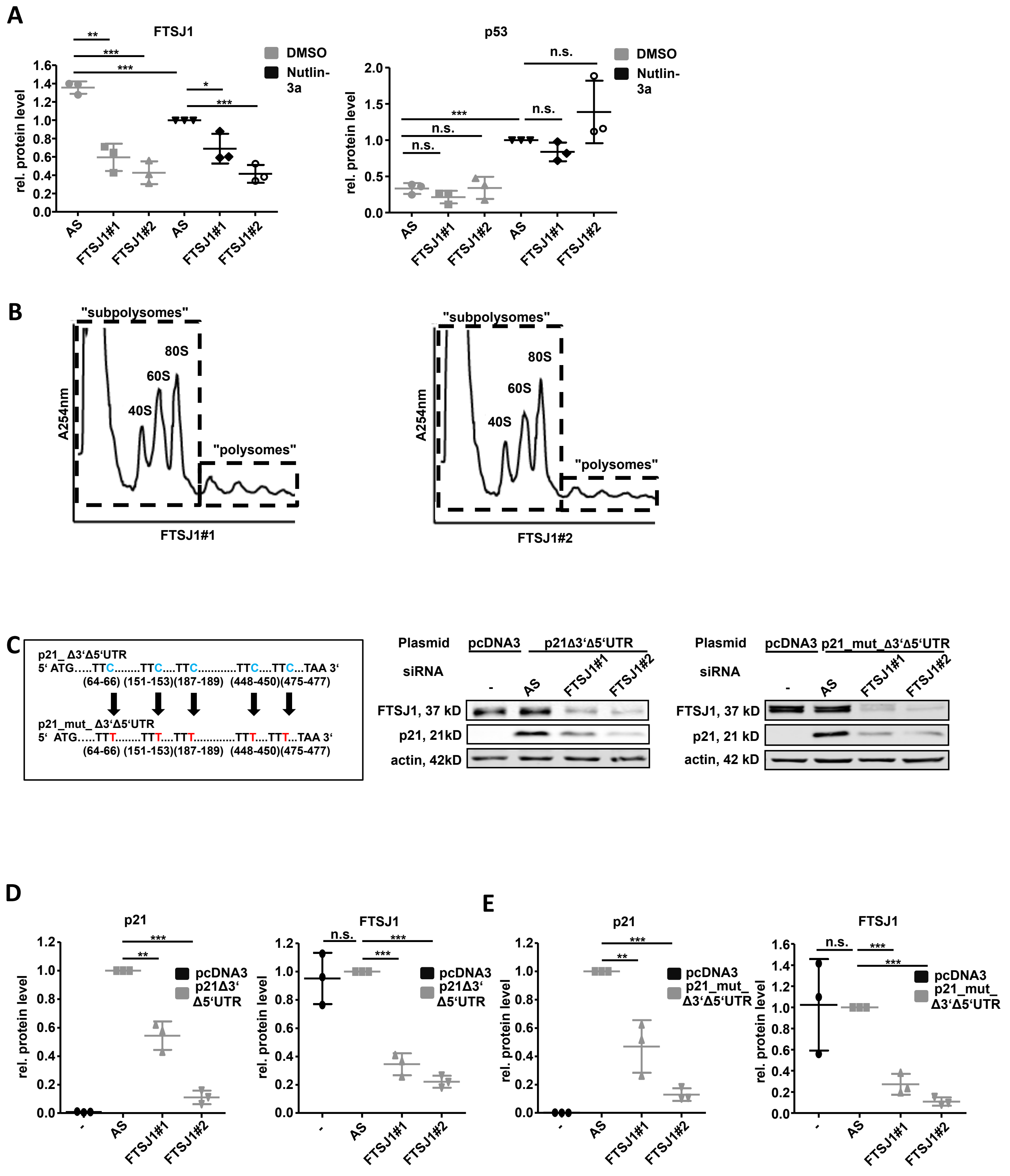

Supplementary Figure 4 A) Densitometric analyses of FTSJ1 and p53 immunoblots derived from three independent experiments (corresponding to Figure 4A) normalised to the Nutlin-3a control siRNA (AS) condition. **B)** Polyribosome gradients corresponding to Figure 4B. FTSJ1 regulates p21 (*CDKN1A*) mRNA translation independent of the codon usage for phenylalanine^{TTC} **C)** H1299 cells were treated either with control siRNA (AS) or two different FTSJ1 siRNAs (FTSJ#1 and FTSJ1#2) for 72h and co-transfected either with a control vector (pcDNA3) or with a p21 expression construct with the regular TTC codon usage for phenylalanine and lacking the 3'UTR and the 5'UTR (p21Δ3'Δ5'UTR) (upper left and middle panel) or a p21 expression construct with alternative codon usage (TTT) for phenylalanine (p21_mut_Δ3'Δ5'UTR) (lower left and right panel). Cell extracts were analysed by immunoblotting with the indicated antibodies. **D)** Densitometric analyses of p21 (left panel) and FTSJ1 (right panel) immunoblots derived from three independent experiments normalised to the control siRNA (AS) condition corresponding to C (middle panel). **E)** Densitometric analyses of p21 (left panel) and FTSJ1 (right panel) immunoblots derived from three independent experiments normalised to the control siRNA (AS) condition corresponding to C (right panel)..

*p < 0.05, **p < 0.01, ***p < 0.001 (Student's-t-test); Data are presented as mean ± stdv. Source data are provided as a Source Data file.

Supplementary Figure 5

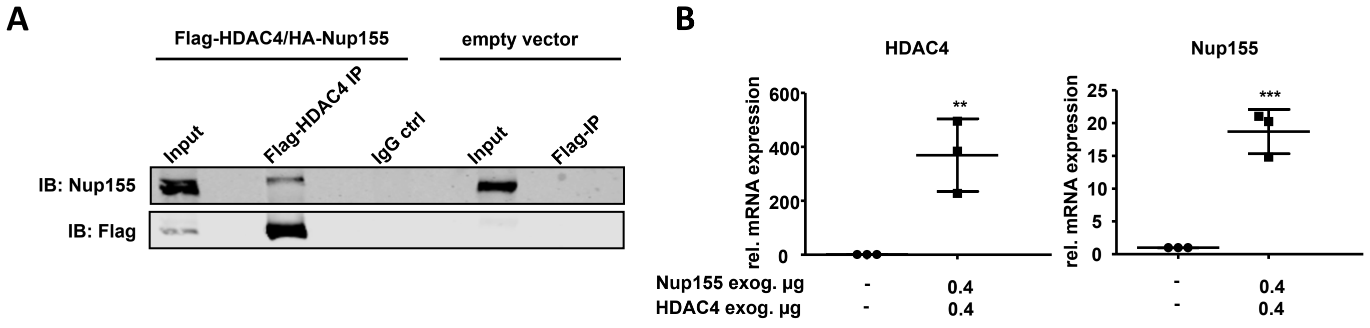

Supplementary Figure 5 A HepG2 cells were co-transfected with flag tagged HDAC4 and HA-tagged Nup155 or an empty vector control. Immunoprecipitation was performed using anti-flag antibody or IgG control. Immunoblotting (IB) was performed with indicated antibodies. **B)** Relative *HDAC4* mRNA (left panel) or *NUP155* mRNA (right panel) expression as measured by qRT-PCR for Nup155-HA and HDAC4-Flag co-transfected cells (0.4 µg each) compared to the empty vector control (-). Data are derived from three independent experiments and normalised to the empty vector control condition.

**p < 0.01, ***p < 0.001 (Student's-t-test); Data are presented as mean ± stdv. Source data are provided as a Source Data file.

Supplementary Figure 6

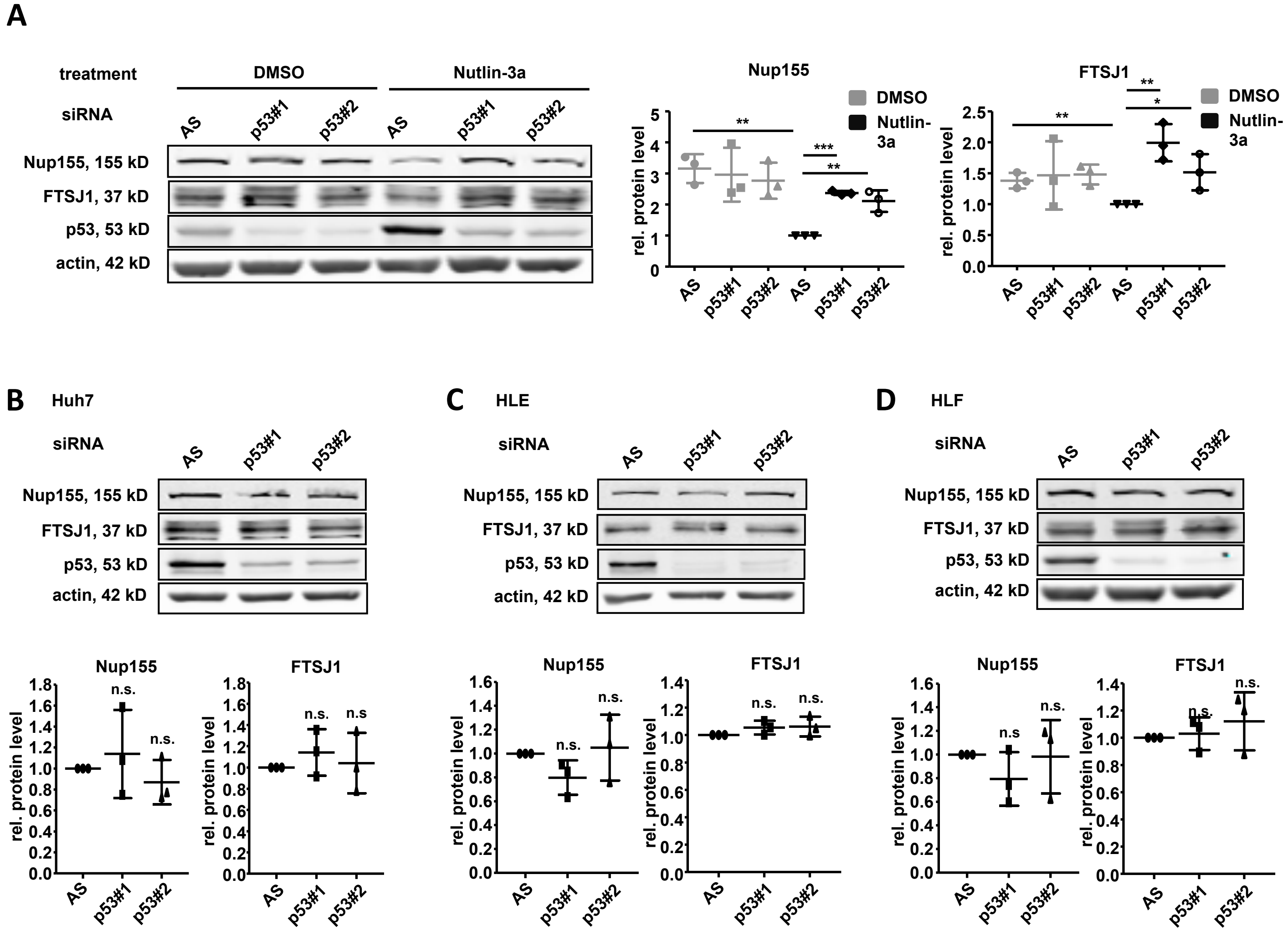

Supplementary Figure 6 A) Sk-Hep1 cells were treated either with control siRNA (AS) or two different p53 siRNAs (p53#1 and p53#2) for 72h. Cells were harvested 48h after Nutlin-3a treatment and extracts were analysed by immunoblotting with the indicated antibodies (left panel). Densitometric analyses of Nup155 and FTSJ1 immunoblots derived from three independent experiments (middle and right panel) normalised to the Nutlin-3a control siRNA (AS) condition. **B-D**) Huh7 cells (B) HLE cells (C), and HLF cells (D) were treated either with control siRNA (AS) or two different p53 siRNAs (p53#1 and p53#2). Cells were harvested 96h after siRNA treatment and extracts were analysed by immunoblotting with the indicated antibodies (upper panels). Densitometric analyses of Nup155 and FTSJ1 immunoblots derived from three independent experiments (lower panels) normalised to the control siRNA (AS) condition.

Supplementary Figure 7

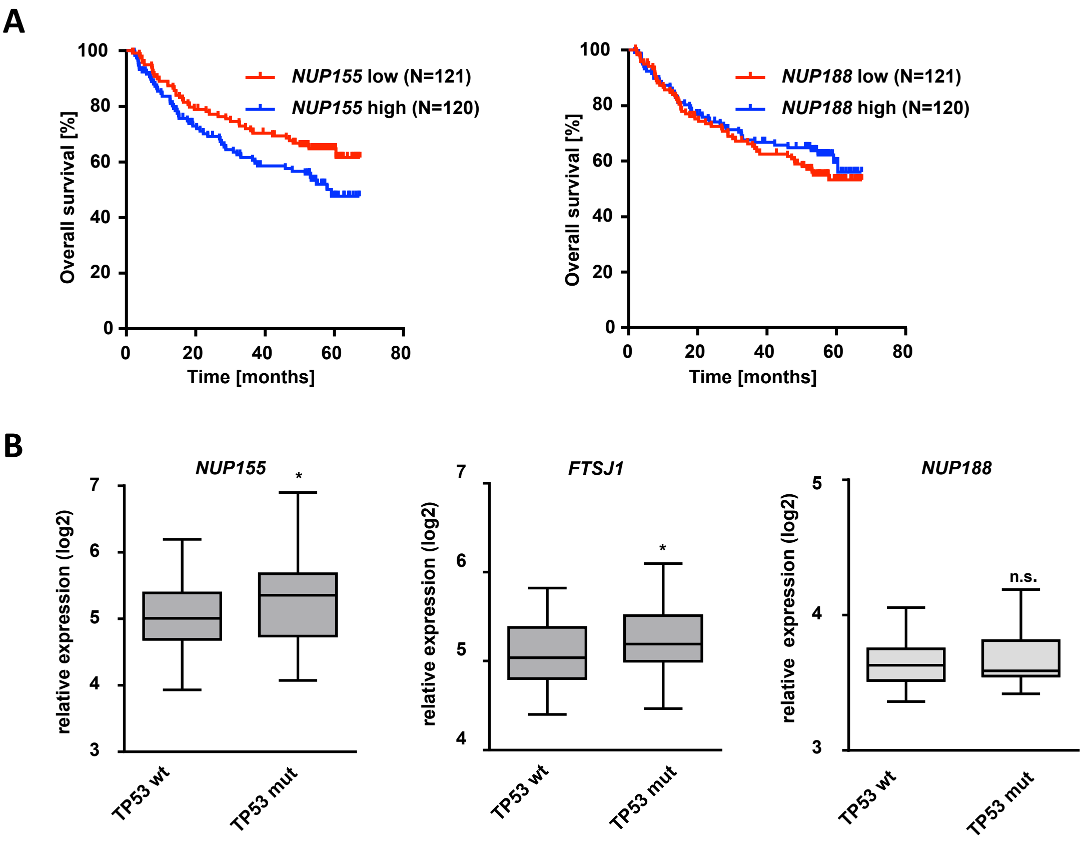

Supplementary Figure 7 A) Kaplan-Meier-Curves show overall survival data of HCC patients with higher or lower than median expression of *NUP155* (left panel, log-rank p = 0.047) or *NUP188* (right panel, log-rank p = 0.423) transcript levels. **B)** Relative transcript levels of *NUP155* (left panel), *FTSJ1* (middle panel), and *NUP188* (right panel) in HCC patient samples with wild-type p53 (TP53 wt, n = 132) or mutant p53 (TP53 mut, n = 46). *p < 0.05, **p < 0.01, ***p < 0.001 (Student's-t-test); The center line of the box plots denotes the median, the bounds of the box indicate the 25th to 75th percentiles and the whiskers represent the 5th to 95th percentiles, respectively. Source data are provided as a Source Data file.

Supplementary Table 1: p53 targets

Ratio.H.L.normalized	average_fdrtool.qval	id	description	short.name
0.40126	5.79E-05	Q9ULZ3	Apoptosis-associated speck-like protein containing a CARD	ASC_HUMAN
1.7524	0.02738253	P49815	Tuberin	TSC2_HUMAN
0.55595	0.041334485	P38936	Cyclin-dependent kinase inhibitor 1	CDN1A_HUMAN
0.56551	0.051893348	P07339	Cathepsin D heavy chain	CATD_HUMAN
1.5738	0.12487226	P39060	Endostatin	COIA1_HUMAN
1.5378	0.163358106	Q92597	Protein NDRG1	NDRG1_HUMAN
1.5033	0.208990773	O43715	TP53-regulated inhibitor of apoptosis 1	TRIA1_HUMAN
0.6321	0.220069892	Q92466	DNA damage-binding protein 2	DDB2_HUMAN
0.647	0.271338602	P22570	NADPH:adrenodoxin oxidoreductase, mitochondrial	ADRO_HUMAN
0.65949	0.323697826	Q53FA7	Quinone oxidoreductase PIG3	QORX_HUMAN
0.67001	0.375619484	Q7LG56	Ribonucleoside-diphosphate reductase subunit M2 B	RIR2B_HUMAN
0.68288	0.439556063	Q99856	AT-rich interactive domain-containing protein 3A	ARI3A_HUMAN
1.3824	0.444170874	O14727	Apoptotic protease-activating factor 1	APAF_HUMAN
0.69515	0.498943251	Q07812	Apoptosis regulator BAX	BAX_HUMAN
0.72685	0.634991986	P62736;P68032;P68133;P63267	Actin, aortic smooth muscle	ACTA_HUMAN
0.74917	0.710975786	Q92851	Caspase-10 subunit p23/17	CASPA_HUMAN
0.75749	0.735027043	Q08257	Quinone oxidoreductase	QOR_HUMAN
1.2299	0.763800312	P55212	Caspase-6 subunit p11	CASP6_HUMAN
0.77628	0.78157486	Q99988	Growth/differentiation factor 15	GDF15_HUMAN
0.78719	0.804171015	Q658P3	Metalloreductase STEAP3	STEA3_HUMAN
1.1923	0.814747589	Q00987	E3 ubiquitin-protein ligase Mdm2	MDM2_HUMAN
1.1868	0.821234519	O14763	Tumor necrosis factor receptor superfamily member 10B	TR10B_HUMAN
0.79674	0.821615074	Q9NQ88	Fructose-2,6-bisphosphatase TIGAR	TIGAR_HUMAN
1.1765	0.832762626	P06400	Retinoblastoma-associated protein	RB_HUMAN
1.1722	0.837343109	Q03518	Antigen peptide transporter 1	TAP1_HUMAN
0.82922	0.867619152	P26358	DNA (cytosine-5)-methyltransferase 1	DNMT1_HUMAN
0.83279	0.871637634	P08238	Heat shock protein HSP 90-beta	HS90B_HUMAN
1.1065	0.892490004	P05121	Plasminogen activator inhibitor 1	PAI1_HUMAN
1.1027	0.89494212	P43246	DNA mismatch repair protein Msh2	MSH2_HUMAN
0.85886	0.896232922	P04637	Cellular tumor antigen p53	P53_HUMAN
0.86947	0.904237481	P42858	Huntingtin	HD_HUMAN
1.0667	0.915002491	P29590	Protein PML	PML_HUMAN
0.887	0.915467361	P12004	Proliferating cell nuclear antigen	PCNA_HUMAN

0.8889	0.91655381	Q96QD8	Sodium-coupled neutral amino acid transporter 2	S38A2_HUMAN
1.0611	0.917659281	Q9BRQ8	Apoptosis-inducing factor 2	AIFM2_HUMAN
0.90018	0.92254381	Q05397	Focal adhesion kinase 1	FAK1_HUMAN
1.0459	0.924322082	P68104; Q5VTE0	Elongation factor 1-alpha 1	EF1A1_HUMAN
1.0431	0.925466896	P07203	Glutathione peroxidase 1	GPX1_HUMAN
1.0372	0.927799928	P00533	Epidermal growth factor receptor	EGFR_HUMAN
0.91274	0.928383	Q9Y6D9	Mitotic spindle assembly checkpoint protein MAD1	MD1L1_HUMAN
0.91664	0.930038074	Q9Y478	5'-AMP-activated protein kinase subunit beta-1	AAKB1_HUMAN
1.0219	0.933377782	P29317	Ephrin type-A receptor 2	EPHA2_HUMAN
0.93448	0.936781454	Q92696	Geranylgeranyl transferase type-2 subunit alpha	PGTA_HUMAN
0.9372	0.937701767	P17931	Galectin-3	LEG3_HUMAN
0.95273	0.942484736	P54278	Mismatch repair endonuclease PMS2	PMS2_HUMAN
0.95432	0.942932599	P11142	Heat shock cognate 71 kDa protein	HSP7C_HUMAN
0.9656	0.945910594	P31947	14-3-3 protein sigma	1433S_HUMAN
0.96808	0.946521203	P40692	DNA mismatch repair protein Mlh1	MLH1_HUMAN

Supplementary Table 2 NPC components

Ratio.H.L.normalized	average_fdrtool.qval	id	description	short.name
0.23957	1.85E-12	O75694	Nuclear pore complex protein Nup155	NU155_HUMAN
0.72088	0.611772033	P49792	E3 SUMO-protein ligase RanBP2	RBP2_HUMAN
0.72239	0.617759146	Q5SRE5	Nucleoporin NUP188 homolog	NU188_HUMAN
0.7345	0.662960272	P52948	Nuclear pore complex protein Nup98	NUP98_HUMAN
0.77682	0.782765121	Q7Z3B4	Nucleoporin p54	NUP54_HUMAN
0.79175	0.812756919	Q8N1F7	Nuclear pore complex protein Nup93	NUP93_HUMAN
0.80395	0.833484606	Q92621	Nuclear pore complex protein Nup205	NU205_HUMAN
0.83358	0.8725033	Q9NRG9	Aladin	AAAS_HUMAN
0.84229	0.88151253	P37198	Nuclear pore glycoprotein p62	NUP62_HUMAN
0.84961	0.88837916	Q8WYP5	Protein ELYS	ELYS_HUMAN
0.84981	0.88855837	A8CG34; Q96HA1	Nuclear envelope pore membrane protein POM 121C	P121C_HUMAN
0.85476	0.892859047	Q9BW27	Nuclear pore complex protein Nup85	NUP85_HUMAN
0.90459	0.924687122	P35658	Nuclear pore complex protein Nup214	NU214_HUMAN
0.90867	0.926578797	Q96EE3	Nucleoporin SEH1	SEH1_HUMAN
0.91002	0.927186203	P49790	Nuclear pore complex protein Nup153	NU153_HUMAN
0.91138	0.927789063	P12270	Nucleoprotein TPR	TPR_HUMAN
0.91951	0.931211984	Q8TEM1	Nuclear pore membrane glycoprotein 210	PO210_HUMAN
0.92527	0.933461017	P55735	Protein SEC13 homolog	SEC13_HUMAN
0.93065	0.935439976	Q12769	Nuclear pore complex protein Nup160	NU160_HUMAN
0.94451	0.940049108	Q8WUM0	Nuclear pore complex protein Nup133	NU133_HUMAN
0.94463	0.940086171	P78406	mRNA export factor	RAE1L_HUMAN
0.97787	0.946183809	Q9BTX1	Nucleoporin NDC1	NDC1_HUMAN
0.9901	0.943055559	Q8NFH4	Nucleoporin Nup37	NUP37_HUMAN
0.99862	0.940692707	P57740	Nuclear pore complex protein Nup107	NU107_HUMAN
1.0011	0.939974922	Q99567	Nuclear pore complex protein Nup88	NUP88_HUMAN
1.1338	0.872691561	P52594	Arf-GAP domain and FG repeat-containing protein 1	AGFG1_HUMAN
1.1492	0.859657983	O15504	Nucleoporin-like protein 2	NUPL2_HUMAN
1.1686	0.841075697	Q9UKX7	Nuclear pore complex protein Nup50	NUP50_HUMAN

Supplementary Table 3: Sequences of transfected siRNAs.

siRNA	sequence
Nup155#1	UUCAGUAAUGCUGCUCGUGUA
Nup155#2	GCCGGUUAUUCAGACCCUAUA
Nup155#3	CCCACCGUUUCUGGCAUGUCA
Drosha#1	AAGGGAUUAACACCUUGAUAA
Drosha#2	AUCGAUCAACUGGAUCGUGAA
Nup35#1	CCGCGUUAGGAUCUGAACCAA
Nup35#2	CAGCCACGAAAGACGACAUUA
Nup93#1	CAGAUGUGACGUCACCGACAA
Nup93#2	GCGCGGCAAAUUUAUAUCUAU
Nup188#1	AUGGGCUUAUUUGUCAGGAUA
Nup188#2	GCAGUGGAAGAGAGAGUUA
FTSJ1#1	CAACGAGGCCUGGACAAACAA
FTSJ1#2	CUCCAUGAUGUUGAUGAGUAU
Z385A#1	CUCCCUGACAAUAAAGUCUGA
Z385A#2	AACUCGGAGGUCCAACUGAAA
RL7L1#1	AAGGUGCGUCUCAGACGACUA
RL7L1#2	CUGCGUAUAGUGGAACCUUAU
NOG1#1	UAGACACGUGUUUGAUAUGAA
NOG1#2	UACGGUGGUGCCGUCCGCCAA
SEN15#1	GUGGGAGAGAAUCAACCGUAA
SEN15#2	CUGGGUCUAGGAAAUAGUCAA
p53#1	UGUUCCGAGAGCUGAAUGA
p53#2	AAGGAAAUUUGCGUGUGGAGU
p21#1	CAGUUUGUGUGUCUUAAUUAU
p21#2	CUGGCAUUAGAAUUAUUUAAA
HDAC4#1	UUCCAAUGUAUUCCAAGCUAA
HDAC4#2	CCGGCGUGGGUUUCAACGUCA

Supplementary Table 4: Sequences of primers used for qRT-PCR.

primer	sequence
Nup155#F	AAAATGCTGGACGGCTCATC
Nup155#R	CCATAAGCAGCTCGGAAAGGT
Nup155#intex_F	TGACACAATTTGCCTCACATTTCA
Nup155#intex_R	CCAAAAGGTGCAGTGGCTTC
p21#F	GGCGGCAGACCAGCATGACAGATT
p21#R	GCAGGGGGCGGCCAGGGTAT
L32#F	TTCCGGTCCACAACGTCAAG
L32#R	TGTGAGCGATCTCGGCAC
FTSJ1#F	AAACCCCTGCTGGACCATTC
FTSJ1#R	TCATAGGAGCTCAGGTCCCC
FTSJ1#intex_F	AGTTTCGCCAAATATGTGAGTC
FTSJ1#intex_R	TGGGTTAAGGTGAACAACTCAT
Tubb5#F	ATTTCTTTATGCCTGGCTTTGC
Tubb5#R	TTCTTGGCATCGAAGACCTG
Fluc#F	CCTCTGGATCTACTGGGTTACCTAAG
Fluc#R	TCTGGCATGCGAGAATCTGA
Rluc#F	GAATTTGCAGCATATCTTGAACCAT
Rluc#R	GGATTTCACGAGGCCATGATAA
HDAC4#F	CCTCACTCCCTACCTGAGCA
HDAC4#R	CCCAGGCCTGTGACGA
