# Supplementary material for: Nucleoporin Nup155 is part of the p53 network in liver cancer
Source: Nat Commun. 2019 May 14;10:2147. doi: 10.1038/s41467-019-10133-z (PMC6517424; doi:10.1038/s41467-019-10133-z)
Supplement: Supplementary file 3 — Description of Additional Supplementary Files [file 41467_2019_10133_MOESM3_ESM.docx]

**Description of Supplementary Files**

**File Name:** Supplementary Data 1

**Description:** Proteins quantified by LC/MS-MS (corresponding to Figure 1A) Ratio.H.L.normalized = Ratio of dimethyl labled peptides (heavy vs light, Nup 155 kd vs Ctrl) normalized to the total number of peptides used for each protein group quantification in each condition average_fdrtool.qval = Statistical measure for significance. Calculated using the R-package"fdrtool" based on the Benjamini-Hochberg procedure. Protein groups with q-value < 0.1 were considered as significantly affected by the depletion of Nup155.
