# Supplementary material for: Nucleoporin Nup155 is part of the p53 network in liver cancer
Source: Nat Commun. 2019 May 14;10:2147. doi: 10.1038/s41467-019-10133-z (PMC6517424; doi:10.1038/s41467-019-10133-z)
Supplement: Supplementary file 5 — Reporting Summary [file 41467_2019_10133_MOESM5_ESM.pdf]

nature research

Corresponding author(s): Stephan Singer

Last updated by author(s): Jan 29, 2019

Reporting Summary

Nature Research wishes to improve the reproducibility of the work that we publish. This form provides structure for consistency and transparency in reporting. For further information on Nature Research policies, see Authors & Referees and the Editorial Policy Checklist.

Statistics

For all statistical analyses, confirm that the following items are present in the figure legend, table legend, main text, or Methods section.

n/a	Confirmed	
☐	☒	The exact sample size (n) for each experimental group/condition, given as a discrete number and unit of measurement
☐	☒	A statement on whether measurements were taken from distinct samples or whether the same sample was measured repeatedly
☐	☒	The statistical test(s) used AND whether they are one- or two-sided<br>*Only common tests should be described solely by name; describe more complex techniques in the Methods section.*
☒	☐	A description of all covariates tested
☐	☒	A description of any assumptions or corrections, such as tests of normality and adjustment for multiple comparisons
☐	☒	A full description of the statistical parameters including central tendency (e.g. means) or other basic estimates (e.g. regression coefficient) AND variation (e.g. standard deviation) or associated estimates of uncertainty (e.g. confidence intervals)
☐	☒	For null hypothesis testing, the test statistic (e.g. F, t, r) with confidence intervals, effect sizes, degrees of freedom and P value noted<br>*Give P values as exact values whenever suitable.*
☒	☐	For Bayesian analysis, information on the choice of priors and Markov chain Monte Carlo settings
☒	☐	For hierarchical and complex designs, identification of the appropriate level for tests and full reporting of outcomes
☒	☐	Estimates of effect sizes (e.g. Cohen's d, Pearson's r), indicating how they were calculated

Our web collection on statistics for biologists contains articles on many of the points above.

Software and code

Policy information about availability of computer code

Data collection	Image Studio Software (v2.1.10, LI-COR Bioscience, Bad Homburg, Germany), Quantity one 1-D Analysis Software (Bio-Rad Laboratories GmbH, München, Germany), StepOne Software Real-Time PCR System (v2.3 ThermoFisher Scientific, Offenbach, Germany), MaxQuant v1.3.0.5, PeakTrak (Version 1.07, Teledyne ISCO, Lincoln NE, USA), Rotor-Gene Q Series Software (v2.3.1 Qiagen, Hilden, Germany), NIS-Elements (F 4.51.00, Nikon Corporation, Minato, Japan).
Data analysis	Excel 2010 (Microsoft, Redmond, WA, USA), GraphPad Prism 6 (GraphPad Software, Inc, La Jolla, CA, USA), R (v2.14.2), Image Studio Software (v2.1.10, LI-COR Bioscience, Bad Homburg, Germany), Quantity one 1-D Analysis Software (Bio-Rad Laboratories GmbH, München, Germany).

For manuscripts utilizing custom algorithms or software that are central to the research but not yet described in published literature, software must be made available to editors/reviewers. We strongly encourage code deposition in a community repository (e.g. GitHub). See the Nature Research guidelines for submitting code & software for further information.

Data

Policy information about availability of data

All manuscripts must include a data availability statement. This statement should provide the following information, where applicable:

- Accession codes, unique identifiers, or web links for publicly available datasets
- A list of figures that have associated raw data
- A description of any restrictions on data availability

The mass spectrometry proteomics data have been deposited to the ProteomeXchange Consortium via the PRIDE partner repository with the dataset identifier PXD012373. In this study the Affymetrix U133A2.0 gene expression data set derived from 247 HCC patients was used that is publicly available through Gene Expression Omnibus accession number GSE14520 (http://www.ncbi.nlm.nih.gov/geo).

Field-specific reporting

Please select the one below that is the best fit for your research. If you are not sure, read the appropriate sections before making your selection.

☒ Life sciences ☐ Behavioural & social sciences ☐ Ecological, evolutionary & environmental sciences

For a reference copy of the document with all sections, see nature.com/documents/nr-reporting-summary-flat.pdf

Life sciences study design

All studies must disclose on these points even when the disclosure is negative.

Sample size	Sample sizes were not predetermined by statistical methods. Samples sizes were considered appropriate based on previously published studies and sufficient to reach statistical significance.
Data exclusions	No data were excluded from the analyses.
Replication	Experimental cell line derived findings were replicated successfully and data are presented as mean ± SD derived from biological replicates (except the focused RNAi screen). Technical replicates ensured to minimize variability of qPCRs.
Randomization	No randomization was conducted for the cell line samples or animal samples used in our study.
Blinding	There was no blinding regarding analyses measuring quantitative parameters which we consider as unbiased.

Reporting for specific materials, systems and methods

We require information from authors about some types of materials, experimental systems and methods used in many studies. Here, indicate whether each material, system or method listed is relevant to your study. If you are not sure if a list item applies to your research, read the appropriate section before selecting a response.

Materials & experimental systems

n/a	Involved in the study	
☐	☒	Antibodies
☐	☒	Eukaryotic cell lines
☒	☐	Palaeontology
☐	☒	Animals and other organisms
☒	☐	Human research participants
☒	☐	Clinical data

Methods

n/a	Involved in the study	
☒	☐	ChIP-seq
☒	☐	Flow cytometry
☒	☐	MRI-based neuroimaging

Antibodies

Antibodies used	All antibodies were purchased as follows: anti-Nup155 rabbit polyclonal (dilution 1:500, #R34951, Atlas, Stockholm, Sweden); anti-Nup35 rabbit polyclonal (dilution 1:500, #R07940, Atlas, Stockholm, Sweden); anti-Nup93 rabbit polyclonal (dilution 1:500, #R07068, Atlas, Stockholm, Sweden); anti-Nup188 rabbit polyclonal (dilution 1:1000, NBP1-28748, Novus Biologicals, Littleton, US); anti-p21 rabbit polyclonal (dilution 1:250, sc-397, Santa Cruz, Heidelberg, Germany); anti-p21 mouse monoclonal (dilution 1:250, sc-6246, Santa Cruz, Heidelberg, Germany); anti-p53 mouse monoclonal (dilution 1:10000, #554293, Pharmingen/BD Transductional Laboratories, Heidelberg, Germany); anti-p53 rabbit polyclonal (dilution 1:500, sc-6243, Santa Cruz, Heidelberg, Germany); anti-mdm2 mouse monoclonal (dilution 1:250, sc-965, Santa Cruz, Heidelberg, Germany); anti-GFP mouse monoclonal (dilution 1:200, sc-9996, Santa Cruz, Heidelberg, Germany); anti-FTSJ1 rabbit polyclonal (dilution 1:500, PA5-35886, Thermo Scientific, Offenbach, Germany); anti-Drosha mouse monoclonal (dilution 1:200, sc-393591, Santa Cruz, Heidelberg, Germany); anti-Flag mouse monoclonal (dilution 1:3000, F1804, Sigma-Aldrich, Taufkirchen, Germany); anti-HA.11 mouse monoclonal (dilution 1:500, #MMS-101R, Covance, Princeton, NJ, USA); anti-HDAC4 (dilution 1:200, sc-365093, Santa Cruz, Heidelberg, Germany); anti-actin mouse monoclonal (dilution 1:10000, #691001, MP Biomedicals, Illkirch, France) anti-vinculin mouse monoclonal antibody (dilution 1:5000, V9131, Sigma-Aldrich, Taufkirchen, Germany). Fluorescent-secondary antibodies (LI-COR Bioscience, Bad Homburg, Germany); secondary antibodies (1:2000 horseradish peroxidase goat anti-mouse ab6789 and horseradish peroxidase goat anti-rabbit ab6721, Abcam, Cambridge, UK).
Validation	All antibodies were commercially available and validation included information of the providers and/or siRNA mediated knockdown experiments as well as size control by western blot bands.

Eukaryotic cell lines

Policy information about cell lines

Cell line source(s)	The cell lines H1299, HepG2 and SkHep1 were purchased from the ATCC (American Type Culture Collection, Manassas, USA). The cell lines Huh7, HLE and HLF were purchased from the JCRB Cell Bank (Japanese Collection of Research Bioresources Cell Bank). The Hep3B-4Bv (expressing the temperature-sensitive p53val135 mutation) were kindly provided by M. Oren. The H1299 derivative cell line expressing p21 from an artificial Tetracycline (Tet)-sensitive cDNA construct (H24-p21) was kindly provided by C. Prives.
Authentication	The cell lines HepG2, SkHep1, HLE, HLF, H1299 and HuH7 have been validated using STR-analyses (Leibniz-Institut, DSMZ, Braunschweig, Germany).
Mycoplasma contamination	All cell lines have regularly been tested for mycoplasma contamination using the MycoAlertTM Plus Mycoplasma Detection Kit (LT07, Lonza, Cologne, Germany).
Commonly misidentified lines (See ICLAC register)	There are no misidentified cell lines in this study.

Animals and other organisms

Policy information about studies involving animals; ARRIVE guidelines recommended for reporting animal research

Laboratory animals	The laboratory of L. Zender kindly provided the murine HCC samples which were generated as described previously. Dauch et al. Nature medicine 2016, 22(7): 744-753.
Wild animals	The study did not involve wild animals.
Field-collected samples	The study did not involve samples collected from field.
Ethics oversight	The laboratory of L. Zender kindly provided the murine HCC samples which were generated as described previously. Dauch et al. Nature medicine 2016, 22(7): 744-753.

Note that full information on the approval of the study protocol must also be provided in the manuscript.
